# Smoking and SARS-CoV-2 Disease (COVID-19): Dangerous Liaisons or Confusing Relationships?

**DOI:** 10.3390/jcm9051321

**Published:** 2020-05-02

**Authors:** Giovanni Li Volti, Massimo Caruso, Riccardo Polosa

**Affiliations:** 1 Department of Biomedical and Biotechnological Sciences, University of Catania, Via S. Sofia, 97 95131 Catania, Italy; mascaru@unict.it; 2 Center of Excellence for the acceleration of Harm reduction - CoEHAR; University of Catania, Via S. Sofia, 97 95131 Catania, Italy; polosa@unict.it; 3 Department of Clinical and Experimental Medicine; University of Catania, Via S. Sofia, 97 95131 Catania, Italy

**Keywords:** COVID-19, SARS-Cov-2, smoking, angiotensin-converting enzyme-2

We read with great interest the article by Brake SJ and colleagues [1] investigating the relationship between smoking and angiotensin-converting enzyme-2 (ACE-2) and the potential implication for COVID-19. The authors present findings linking ACE-2 expression to smoking in a variety of experimental models together with observations of their own; immunohistochemistry data showing an increased expression of ACE-2 in a series of biopsies from a group of current smokers with chronic obstructive pulmonary disease when compared to a control group. The authors then venture into reporting existing Chinese case reports to support their hypothesis that smoking could increase the risk of COVID-19 via upregulation of ACE-2 expression, a known cellular entry gateway for SARS-CoV-2 [2]. However, there are a number of problems with their hypothesis. First, the virus spike protein responsible for ACE-2 binding requires its counterpart to be localized on the plasma membrane in order to be subsequently internalized [3,4]. Therefore, the mere total protein or gene expression is not conclusive to suggest a possible increased virus infection risk. Second, it is known that ACE-2 expression is down regulated on plasma membranes following SARS-CoV-2 infection because of successive internalization of ACE-2-virus complex [5]. Third, simple ACE-2 expression on plasma membranes may be not a conclusive element in order to establish a potential risk factor for virus infection. In fact, once the spike protein is bound to ACE-2, the cell is required to trigger a complex series of biochemical (i.e., activation of specific protease) and molecular signals in order to internalize the virus [3]. In addition, the interplay between COVID-19 and the renin–angiotensin–aldosterone system is complex [6]. The view that overexpression of ACE2 is detrimental does not take into account more recent evidence that up-regulation of ACE2 may in fact be protective against disease severity [7]. Experimental data suggest that infection with SARS-CoV and SARS-CoV-2 leads to down-regulation of ACE2, and this downregulation is harmful due to uncontrolled ACE and angiotensin II activity [2,7]. It has been observed that decreased ACE2 availability contributes to lung injury and ARDS development [8,9]. Therefore, higher ACE2 expression, while seemingly paradoxical, may protect against acute lung injury caused by COVID-19 [10]. To the best of our knowledge, there are no experimental or clinical evidence establishing the potential impact of smoking on the above-described complex mechanisms, some of which remain still elusive. Consistently, several recent clinical and demographical evidence further support the idea that the impact of smoking and risk of SARS-CoV-2 infection is still an open question and a matter of debate. In a recent systematic review of 13 Chinese studies, smoking is vastly protective for hospitalized COVID-19 and similar findings have been now noted in the US [11]. The Centers for Disease Control and Prevention (CDC) [12] report an unusually low prevalence of current smoking among COVID-19 cases (1.3%) compared to the population smoking prevalence in the US (16.5%) [13]. A cross-sectional analysis of 4103 laboratory-confirmed COVID-19 patients treated at academic hospitals in New York City demonstrated again a low smoking prevalence (5.2%) [14].

Consistent with the findings of Farsalinos et al. [11] and CDC [12], the multivariate analysis performed by the New York researchers showed a significant protective effect against hospitalization for current and former tobacco use (OR = 0.71, 95% CI 0.57–0.87 *p* = 0.001). Moreover, smoking was not a risk factor for critical disease or death. Finally, the authors stated that electronic cigarettes and “heat-not-burn” devices are not “safer” than cigarettes since they are still tobacco products producing vapor or smoke and therefore, similarly could cause infectious lung damage as we see with traditional cigarettes. Such statements are highly inaccurate; UK and US health authorities have stated that combustion free tobacco products are less harmful than combustible cigarettes [15,16]. Last but not least, to date, no data or research on vaping and COVID-19 is available. The assertions made by the authors on vaping and COVID-19 are pure speculation.

The complex interaction between smoking and RAAS/ACE-2 poses multiple challenges for the researcher, the clinician and the COVID-19 patient. The jury is still out, and the relationship between smoking and COVID-19 should be carefully investigated.
